# The effects of probiotic supplementation on cardiometabolic health in patients with prediabetes: a systematic review, meta-analysis, and GRADE assessment

**DOI:** 10.3389/fnut.2025.1616476

**Published:** 2025-09-01

**Authors:** Rongfang Liu, Gao Wong

**Affiliations:** Shenzhen TCM Anorectal Hospital (Futian), Shenzhen, Guangdong, China

**Keywords:** obesity, body weight, body mass index, meta-analysis, probiotics

## Abstract

**Introduction:**

Previous studies have yielded conflicting results regarding the effect of probiotics on prediabetes. To address this, we did an updated systematic review and meta-analysis of existing studies to evaluate the effects of probiotics on prediabetes.

**Methods:**

We conducted a thorough search for pertinent trials on the impact of probiotic supplementation on prediabetes using various databases such as PubMed, Medline, and Google Scholar.

**Results:**

Ten RCTs were included. Probiotic supplementation significantly reduced HbA1c (WMD = −0.11; 95% CI: −0.18, −0.04; *p* < 0.001; *I*^2^ = 0.0%) and increased HDL-C (WMD: 2.37; 95% CI: 1.02, 3.71; *p* < 0.001; *I^2^* = 0.0%). Moreover, there were no significant effects of probiotic supplementation on FBS, insulin, HOMA-IR, LDL-C, TC, TG, BMI, SBP, and DBP. GRADE assessment showed high for HbA1c and HDL-C and moderate for BMI, SBP, DBP, insulin, HOMA-IR, TC, and LDL-C, and low for FBS and TG.

**Conclusion:**

Probiotic supplementation reduces HbA1c levels and increases HDL-C in individuals with prediabetes. Future research involving large-scale, international RCTs is essential to further validate its therapeutic potential.

## Introduction

Prediabetes occurs when fasting or postprandial blood sugar is elevated, though not enough to meet the criteria for full-blown diabetes (1). Prediabetes carries a significant risk of progression to type 2 diabetes (T2DM) (2). The prevalence of prediabetes is growing in countries at all economic levels, from advanced to emerging economies. In 2019, it was reported that an estimated 373.9 million people, or 7.5% of the global adult population aged 20–79 years, had prediabetes (3). Lifestyle changes and drug treatments both have their limitations and potential side effects in managing prediabetes (4). This highlights the urgent need for natural and safe solutions to control and delay the progression from prediabetes to diabetes (5). Notably, prediabetes is a reversible stage in clinical practice (6, 7). Recent studies have identified specific mechanisms that contribute to the progression from prediabetes to diabetes. Significant microbial changes occur in the gut during this process, impacting intestinal permeability, metabolic control, and insulin resistance mechanisms (8).

Probiotics have beneficial effects on the body by helping to regulate the balance of intestinal microbiota (9). Because of the close connection of gut microbiota to human health and the action of probiotics on gut microbiota, their supplementation can provide good health results (10). Increasing evidence indicates an inverse association between probiotics and hyperglycemia, dyslipidemia, and hypertension (11, 12). Certain probiotic bacterial strains have demonstrated efficacy in enhancing inflammation response, boosting immune function, and postponing the onset of diabetes (13). Several studies have shown the beneficial effects of particular probiotic bacterial strains on glycemic regulation (14–17). Additional metabolic effects, including reduced lipid concentrations, enhanced immune regulation, and diminished oxidative stress, have been found in research on diabetes involving probiotics (18, 19).

A meta-analysis study examined the effect of probiotics on prediabetes (20). First, all these studies were conducted before 2020. As a result of the publication of new clinical trial articles, there is a need to update the findings. Second, none of these studies performed a GRADE assessment, making it impossible to comment on the quality of the obtained evidence. Third, none of these studies have focused on adverse events. An updated review is needed to consolidate the varying results from previous studies regarding the impact of probiotic supplementation on cardiovascular risk factors in individuals with prediabetes. The current meta-analysis of randomized clinical trials (RCTs) aims to provide a comprehensive view of the effects of probiotics on cardiometabolic health in patients with prediabetes.

## Methods

### Study design and protocol registration

The present research was performed in accordance with Preferred Reporting Items for Systematic Reviews and Meta-Analyses (PRISMA) guidelines (21). A detailed protocol outlining the study objectives, inclusion criteria, and analytical methods was registered in the PROSPERO database (CRD42023472957).

### Eligibility criteria

RCTs evaluating the effects of probiotics on cardiometabolic health were included. The PICOS framework was used to determine the inclusion criteria. Participants (P) were individuals with prediabetes, defined as having fasting blood sugar (FBS) concentrations of 100–125 mg/dL, 2-h glucose tolerance test levels of 140–199 mg/dL, or HbA1c between 5.7 and 6.4%. The intervention (I) was probiotic supplementation at any dosage and duration. Comparators (C) included a placebo. Outcomes (O): Primary outcomes; BMI, FBS, HbA1c, insulin, HOMA-IR. Secondary outcomes: systolic blood pressure (SBP), diastolic blood pressure (DBP), low-density lipoprotein cholesterol (LDL-C), total cholesterol (TC), HDL-C, and TG. Study design (S), RCTs were included. Observational studies, review articles, *in vitro* or *in vivo* studies, quasi-experimental studies, and non-randomized trials were excluded.

### Search strategy

Relevant studies published up to August 2024 were searched in the PubMed, Medline, and Google Scholar databases using the following keywords: “probiotics,” “cardiovascular risk factors,” and “randomized controlled trials” (Supplementary material 1). Supplementary searches were conducted in trial registries (e.g., ClinicalTrials.gov) and the references in included articles. Our search was not limited to language or publication date. Both published articles and grey literature were considered.

### Data extraction

The screening process was conducted independently by two researchers, and any disagreements were resolved by a third researcher. The extracted data included study characteristics (author, year, location, and design), participant details (sample size, age, gender, and baseline BMI), intervention specifics (probiotics dosage, duration, and administration method), and outcomes (the mean ± standard deviation (SD) changes of primary outcomes).

We followed the guidelines outlined for data extraction and conversion of quantitative outcomes (22). Continuous outcomes were extracted as means and standard deviations (SDs). When outcomes were reported in different units, they were converted to a uniform scale using the recommended methods in the handbook (23). We contacted the original study authors for clarification of missing or incomplete data. When medians and interquartile ranges (IQRs) were provided instead of means and SDs, we estimated the mean using the formula Mean≈Median and SD ≈ IQR/1.35. If the SD was not available but standard errors (SE) or confidence intervals (CIs) were reported, we calculated the SD using the formulas SD=SE× √n or SD = upper CI bound—lower CI bound/2 × 1.96 for a 95% CI (24). Additionally, for cases where only ranges were provided, we estimated SDs using the formula SD = Range/4, as applicable for normally distributed continuous data (23).

### Risk of bias assessment and GRADE assessment

Two researchers conducted separate assessments to determine the potential for bias in every study. The Cochrane Risk of Bias 2.0 tool (ROB2) was used to evaluate methodological quality (25). Each domain was graded as “low risk,” “some concerns,” or “high risk.” Any differences of opinion were addressed and resolved with a third reviewer. We used the GRADE system to determine the certainty of the evidence for each measured outcome (26). Factors influencing certainty included publication bias, imprecision, indirectness, inconsistency, and risk of bias.

### Statistical analysis

Stata version 14 was used for statistical analyses (StataCorp, College Station, TX, USA), using a random-effects model for pooling the data (27). The overall effect size was calculated using the mean difference (MD) and SDs of changes in the outcome measures, with a correlation coefficient (r) set at 0.8. Meta-analyses were performed when at least three studies reported the same outcome. The weighted mean difference (WMD) and 95% confidence interval (CI) were calculated by combining data from all eligible RCTs (28). To quantify heterogeneity among the selected RCTs, we utilized the *I*^2^ statistic, interpreting results exceeding 50% as indicating considerable heterogeneity (29). Subgroup analysis was conducted to detect possible sources of heterogeneity. Subgroup analysis was also employed to demonstrate the effect size across various subgroups based on age and intervention duration. Sensitivity analyses were performed using the leave-one-out method to determine each study’s influence on the overall findings. Due to fewer than 10 included studies, Begg’s test was used to assess publication bias (30). A significant value was set as <0.05 in all analyses.

## Results

### Characteristics of included studies

**Figure 1:**
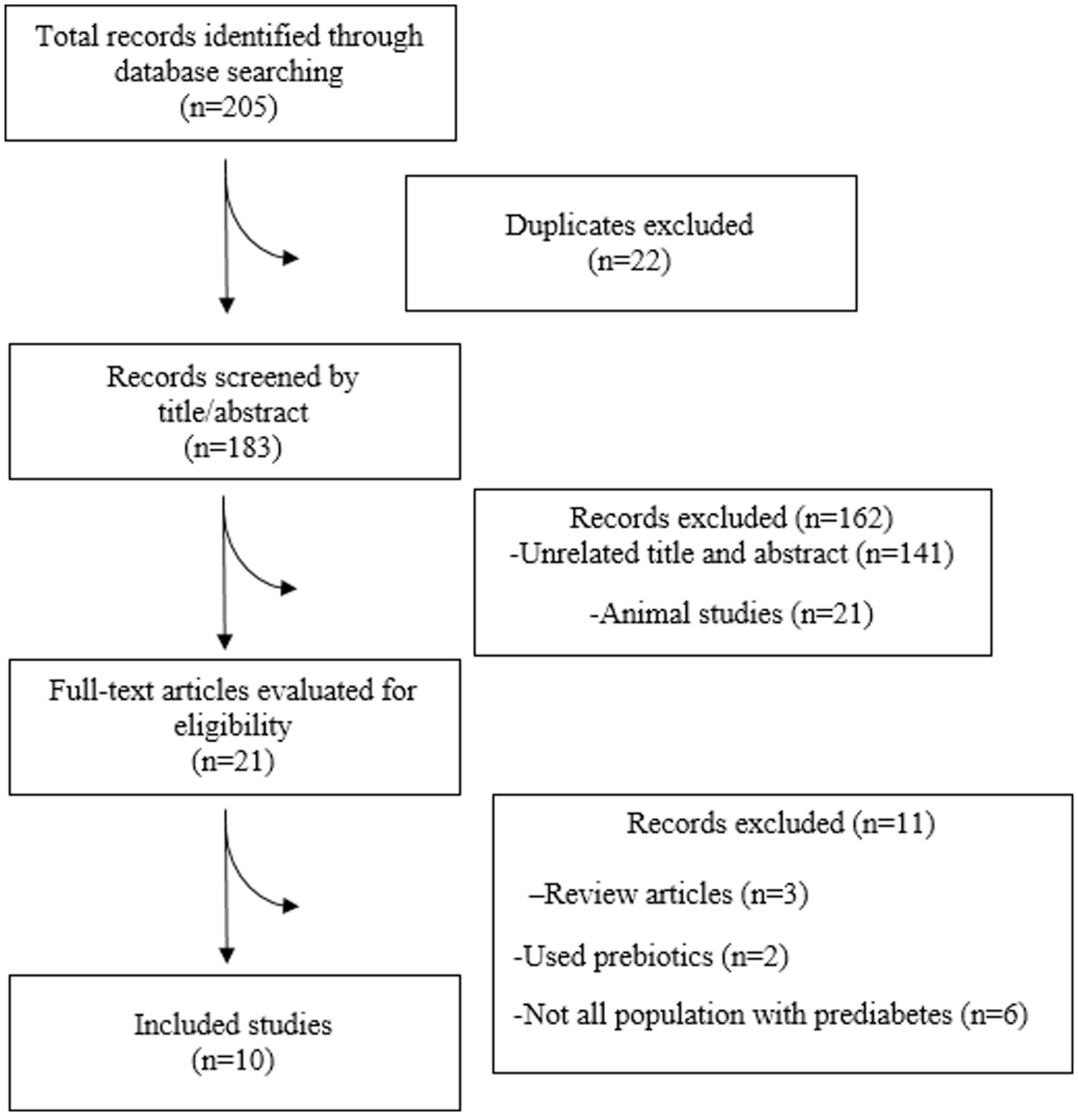
PRISMA flowchart diagram.

In total, 10 RCTs were incorporated into the present systematic review and meta-analysis (31–40) (Figure 1), which included 695 people with prediabetes. Of the 10 RCTs, 4 were conducted in Iran (31, 33–35), 2 in New Zealand (32, 39), 2 in Japan (36, 40), 1 in the Republic of Korea (37), and 1 in Greece (38). Except for one RCT, a double-blind approach was used. The observed sample sizes in the probiotic group ranged from 7 to 76, and in the placebo group, from 10 to 77. The follow-up duration in the RCTs ranged from 8 (35–37) to 24 (32–34) weeks. Five RCTs used capsules as the probiotic dosage form; three RCTs provided the probiotic in powder form, one RCT provided the probiotic in yogurt form, and the remaining RCTs included the probiotic in milk. The bacterial strains used in the included RCTs showed considerable diversity, with *Lactobacillus* and *Bifidobacterium* being the predominant probiotic components. All included RCTs used a placebo as a comparator (31–40). Background information for each assessed study is comprehensively presented in Table 1.

**Table 1:** Baseline characteristics of the included studies.

Study, Country	Sample size	Probiotic strain	Gender/Female (%)	Age (years)	Duration (weeks)	Adverse events Probiotics/Placebo	Quality of study
AkbariRad et al. (31), Iran	35/35	*Lactobacillus, Bifidobacterium, Streptococcus* 500 mg	Both 74.3%	44/43	12	Not reported	High
Barthow et al. (32), New Zealand	76/77	*Lactobacillus* 6 × 10^9^ CFU	Both 53.7%	60/58	24	Nausea (1.4/0%), Stomachache/cramps (5.8/3%), Bloated/swollen stomach (8.5/3%)	High
Kassaian et al. (34), Iran	27/28	*Lactobacillus, Bifidobacterium* 6 × 10^9^ CFU each	Both 52%	53/53	24	Flatulence, dysphagia, and Dyspepsia (7.4/18%)	High
Kassaian et al. (33), Iran	27/28	*Lactobacillus, Bifidobacterium* 6 × 10^9^ CFU each	Both 52%	53/53	24	Flatulence, dysphagia, and Dyspepsia (7/14%)	High
Mahboobi et al. (35), Iran	28/27	*Lactobacillus, Bifidobacterium, Streptococcus* 5.5 × 10^9^ CFU	Both 29.6%	51/50	8	Not reported	High
Naito et al. (36), Japan	48/50	*Lactobacillus* 1 × 10^11^ CFU	Male	46/47	8	Not reported	High
Oh et al. (37), Korea	20/20	*Lactobacillus* 4 × 10^9^ CFU	Both 70%	56/53	8	Non-serious adverse events (15/23.5%)	High
Stefanki et al. (38), Greece	7/10	*Lactobacillus, Bifidobacterium* 45 × 10^9^ CFU	Both 42.9%	15/14	16	Bloating, flatulence, and constipation (prevalence not reported)/Not reported	Low
Tay et al. (39), New Zealand	15/11	*Lactobacillus* 6 × 10^9^ CFU	Both 60%	53/54	12	Mild adverse events: headaches (17%), dizziness/nausea (13%), feeling irritable due to hunger (13%), reduced concentration (4%), increased hunger (4%), feeling grumpy (4%), and general malaise (4%) = did not differ significantly between probiotics vs. placebo	High
Toshimitsu et al. (40), Japan	62/64	*Lactobacillus* 5 × 10^9^ CFU	Both 32.3%	50/51	12	Non-serious adverse events without any significant difference between the groups (16%)	High

### Risk of bias assessment

**Figure 2:**
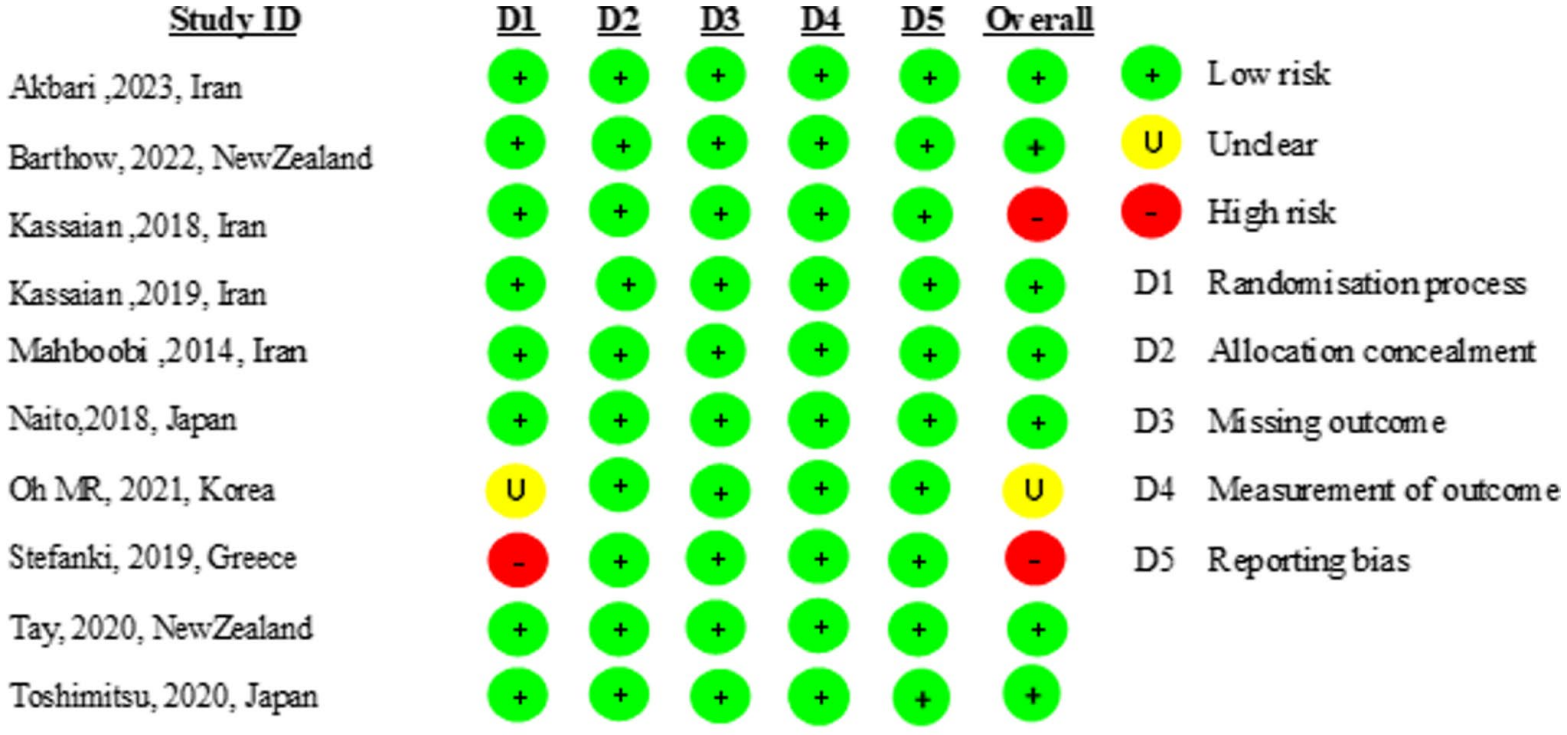
Risk of bias of included studies.

Nine of the 10 included studies were of high quality (31–37, 39, 40). One study did not report the randomization process. The risk of bias is presented in Figure 2.

### Effect of probiotics on glycemic indices

**Figure 3:**
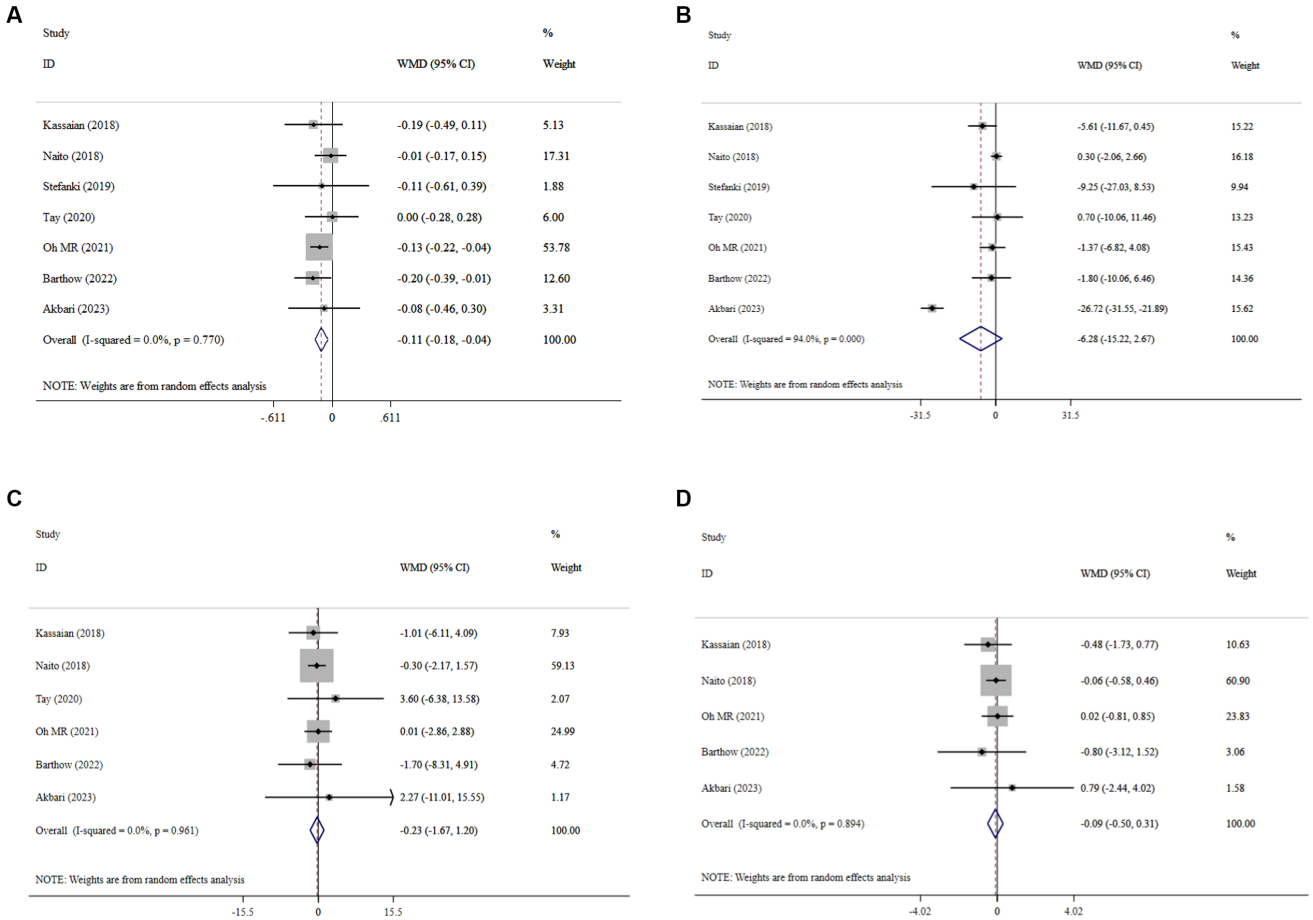
Forest plot details mean difference and 95% confidence intervals (CIs), the effects of probiotic supplementation on HbA1c **(A)**, FBS **(B)**, insulin **(C)**, and HOMA-IR **(D)** levels.

The meta-analysis of comparisons from seven RCTs (n = 441) revealed that probiotic supplementation significantly reduced HbA1c (WMD = −0.11; 95% CI: −0.18, −0.04; *p* < 0.001), with no heterogeneity (*I^2^* = 0.0%, P-heterogeneity = 0.77) (Figure 3A). However, probiotics did not have a significant effect on FBS (WMD = −6.28; 95% CI: −15.22, 2.67; *p* = 0.169; *I^2^* = 94.0%, P-heterogeneity < 0.001) (Figure 3B), insulin (WMD = −0.23; 95% CI: −1.67, 1.20; *p* = 0.749; *I^2^* = 0.0%, P-heterogeneity = 0.931) (Figure 3C), and HOMA-IR (WMD = −0.09; 95% CI: −0.50, 0.31; *p* = 0.649; *I^2^* = 0.0%, P-heterogeneity = 0.894) (Figure 3D) compared to the control group. The results proved robust in sensitivity analyses, with no single trial exerting undue influence on the combined effect size (Supplementary Figures 1–3). However, the overall effects of probiotics on HbA1c were significantly altered when one trial was omitted during sensitivity analysis (WMD = −0.09; 95% CI: −0.19, 0.01; *p* > 0.05) (Supplementary Figure 4) (37). No evidence of publication bias was detected using Begg’s test (*p* > 0.05).

### Effect of probiotics on lipid profile

**Figure 4:**
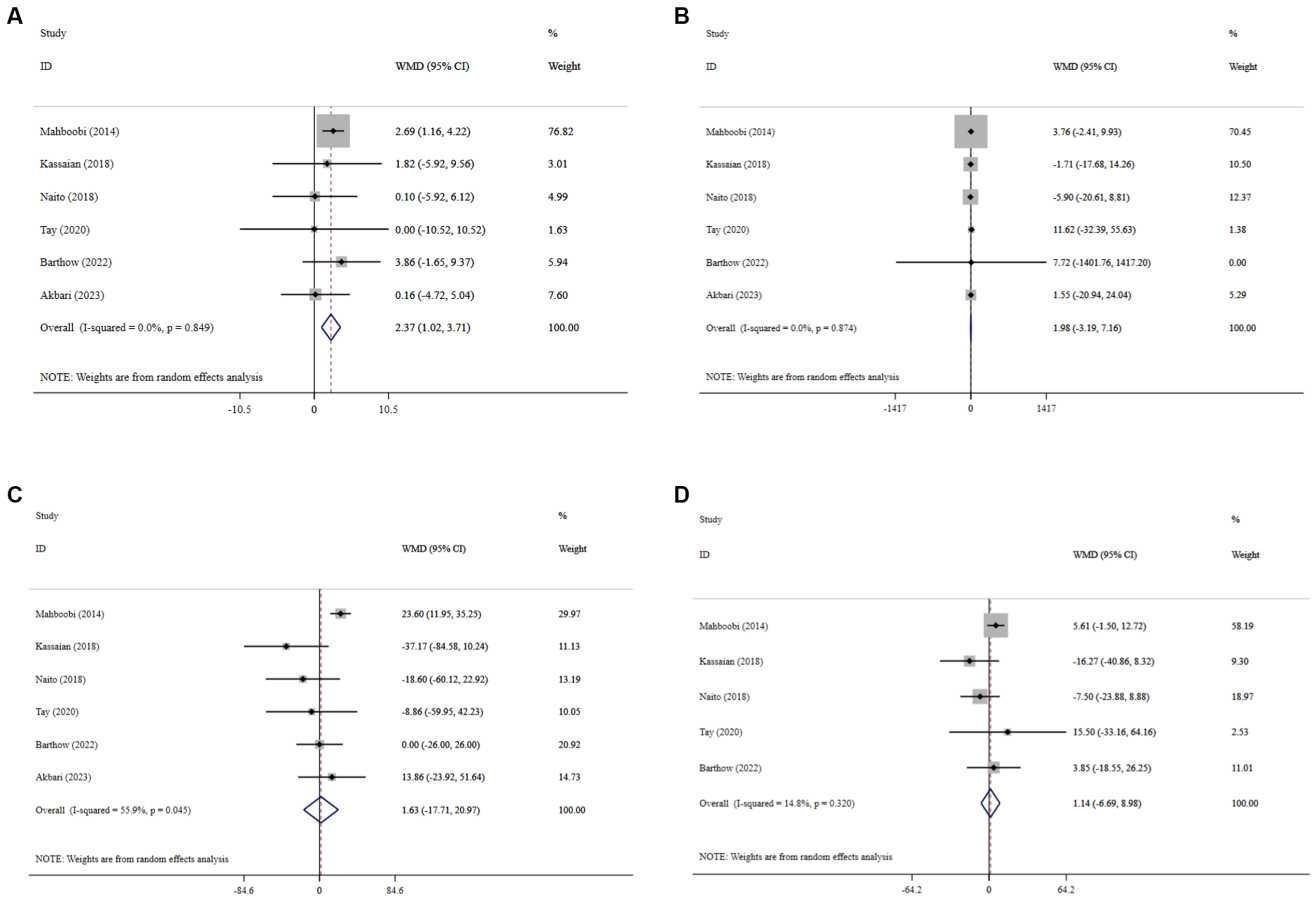
Forest plot details mean difference and 95% confidence intervals (CIs), the effects of probiotic supplementation on HDL-C **(A)**, LDL-C **(B)**, TG **(C)**, and TC **(D)** levels.

Probiotic supplementation significantly increased HDL-C, with a pooled WMD of 2.37 (95% CI: 1.02, 3.71; *p* < 0.001) and without substantial heterogeneity (*I^2^* = 0.0%, P-heterogeneity < 0.849) (Figure 4A). Moreover, probiotics did not have a significant effect on LDL-C (WMD = 1.98; 95% CI: −3.19, 7.16; *p* = 0.453; *I^2^* = 0.0%, P-heterogeneity = 0.874) (Figure 4B), TG (WMD = 1.63; 95% CI: −17.71, 20.97; *p* = 0.869; *I^2^* = 55.9%, P-heterogeneity = 0.045) (Figure 4C), and TC (WMD = 1.14; 95% CI: −6.69, 8.98; *p* = 0.774; *I^2^* = 14.8%, P-heterogeneity = 0.320) (Figure 4D) compared to the control group. Sensitivity analysis revealed that no individual study affected the overall effect size, and confirmed the overall results for the TC, TG, and LDL-C (Supplementary Figures 5–7). However, the overall effects of probiotics on HDL-C changed significantly by excluding the one RCT using sensitivity analysis (WMD = 1.31; 95% CI: −1.49, 4.09; *p* > 0.05) (Supplementary Figure 8) (35). No evidence of publication bias was detected using Begg’s test (*p* > 0.05).

### Effect of probiotics on BMI and blood pressure

**Figure 5:**
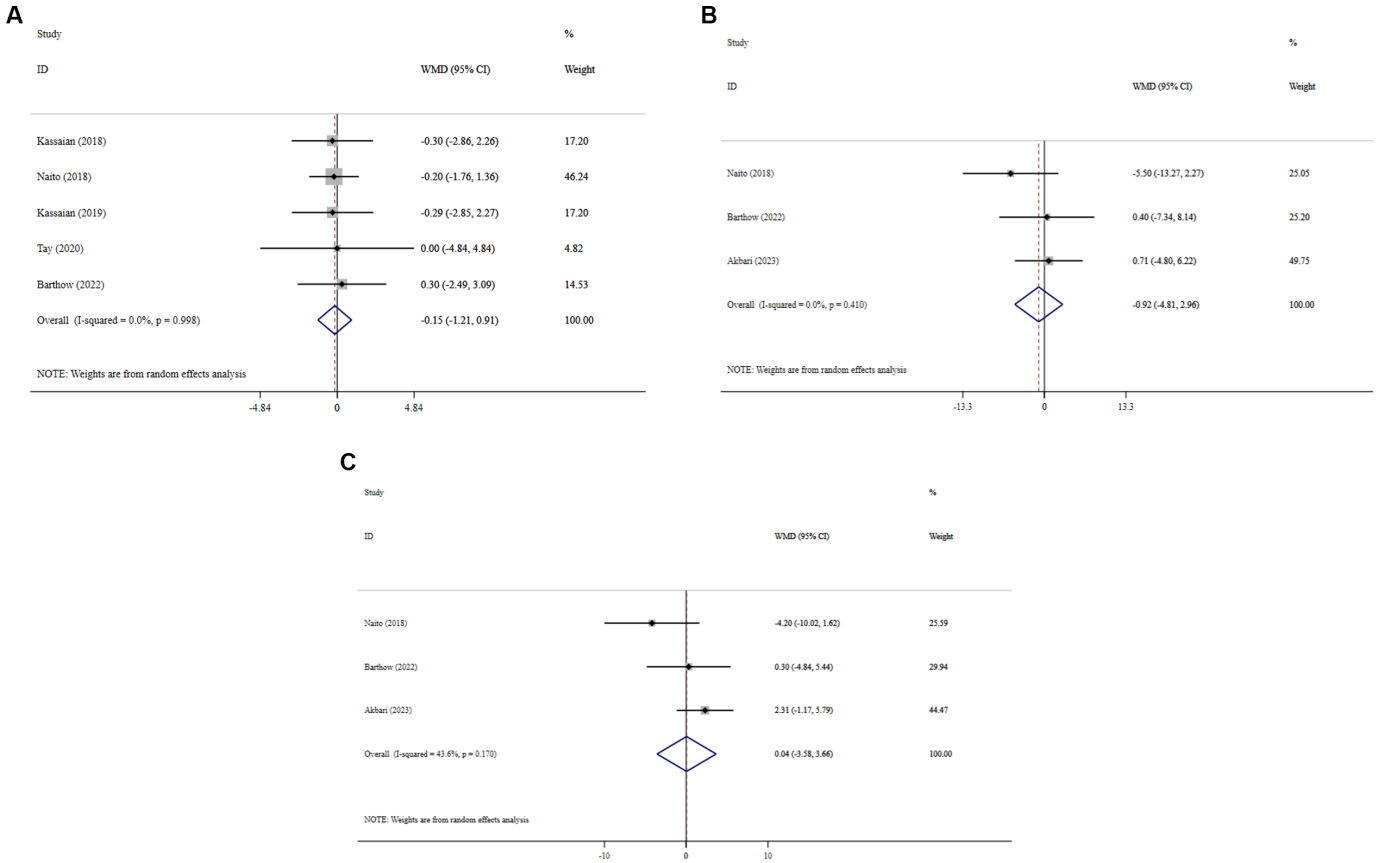
Forest plot detailing mean difference and 95% confidence intervals (CIs), the effects of probiotic supplementation on BMI **(A)**, SBP **(B)**, and DBP **(C)** levels.

Overall, probiotic supplementation did not significantly reduce BMI (WMD = −0.15; 95% CI: −1.21, 0.91; *p* = 0.782; *I^2^* = 0.0%, P-heterogeneity = 0.998) (Figure 5A), SBP (WMD = −0.92; 95% CI: −4.81, 2.96; *p* = 0.641; *I^2^* = 0.0%, P-heterogeneity = 0.410) (Figure 5B), and DBP (WMD = 0.04; 95% CI: −3.58, 3.66; *p* = 0.982; *I^2^* = 43.6%, P-heterogeneity = 0.170) (Figure 5C). Sensitivity analysis showed that excluding any of the trials had no significant impact on the findings (Supplementary Figures 9–11). Begg’s test did not reveal publication bias (*p* > 0.05).

### GRADE assessment

The GRADE assessment revealed that the quality of evidence was high for HbA1c and HDL-C and moderate for HOMA-IR, insulin, BMI, SBP, DBP, TC, and LDL-C, and low for FBS and TG (Table 2).

**Table 2:** Summary of findings and quality of evidence.

Outcome measures	Summary of findings		Quality of evidence assessment (GRADE)					
	No of patients (trials)	WMD (95% CI)	Risk of bias ^a^	Inconsistency ^b^	Indirectness ^c^	Imprecision ^d^	Publication bias ^e^	Quality of evidence ^f^
BMI	363 (5)	−0.15 (−1.21, 0.91)	Not Serious	Not Serious	Not Serious	Serious	Not Serious	Moderate
DBP	306 (3)	−0.92 (−4.81, 2.96)	Not Serious	Not Serious	Not Serious	Serious	Not Serious	Moderate
SBP	306 (3)	0.04 (−3.58, 3.66)	Not Serious	Not Serious	Not Serious	Serious	Not Serious	Moderate
FBS	450 (7)	−6.28 (−15.22, 2.67)	Not Serious	Serious	Not Serious	Serious	Not Serious	Low
HbA1c	671 (7)	−0.11 (−0.18, −0.04)	Not Serious	Not Serious	Not Serious	Not Serious	Not Serious	High
Insulin	424 (6)	−0.23 (−1.67, 1.20)	Not Serious	Not Serious	Not Serious	Serious	Not Serious	Moderate
HOMA-IR	389 (5)	−0.09 (−0.50, 0.31)	Not Serious	Not Serious	Not Serious	Serious	Not Serious	Moderate
TG	433 (6)	1.63 (−17.71, 20.97)	Not Serious	Serious	Not Serious	Serious	Not Serious	Low
TC	363 (5)	1.14 (−6.69, 8.98)	Not Serious	Not Serious	Not Serious	Serious	Not Serious	Moderate
LDL-C	433 (6)	1.98 (−3.19, 7.16)	Not Serious	Not Serious	Not Serious	Serious	Not Serious	Moderate
HDL-C	433 (6)	2.37 (1.02, 3.71)	Not Serious	Not Serious	Not Serious	Not Serious	Not Serious	High

a Risk of bias based on the according to Cochrane risk-of-bias.

b Downgraded if there was a substantial unexplained heterogeneity (I^2^ > 50%, p < 0.10) that was unexplained by meta-regression or subgroup analyses.

c Downgraded if there were factors present relating to the participants, interventions, or outcomes that limited the generalizability of the results. Participants of the included studies were from different health conditions (subgroup analysis was not performed for each disease).

d Downgraded if the 95% confidence interval (95% CI) crossed the minimally important difference (MID) for benefit or harm, and it is downgraded if, the sample size is less than 400 individuals.

e Downgraded if there was an evidence of publication bias using funnel plot.

f Since all included studies were meta-analyses, the certainty of the evidence was graded as high for all outcomes by default and then downgraded based on prespecified criteria. Quality was graded as high, moderate, low, very low.

### Subgroup analysis

When stratified by mean age, studies showed more pronounced improvements in glycemic parameters—especially HbA1c levels—in older populations (>55 years) receiving probiotics versus younger individuals. Additionally, studies with larger sample sizes tended to have more significant results due to higher study power. The duration of intervention significantly influenced outcomes, with probiotic administration for more than 12 weeks demonstrating substantial HbA1c reduction compared to short-term supplementation. While HDL-C increased significantly only in the ≤12-week subgroup, study characteristics (sample size, dosage, and gender) showed no significant heterogeneity for HDL-C or HbA1c outcomes. This indicates time-dependent effects on HbA1c but suggests HDL-C responses may depend on additional factors beyond duration (see Table 3).

**Table 3:** Subgroup analyses for the effects of probiotics supplementation on patients with prediabetes.

	Number	WMD (95% CI)	*I*^2^ (%)	P-heterogeneity
Probiotic on FBS				
Overall	7	−6.28 (−15.22, 2.67)	93.2	<0.001
Age (years)				
≤18	1	−9.25 (−27.03, 8.53)	–	–
18−55	4	−8.02 (−22.31, 6.26)	96.9	<0.001
˃55	2	−1.50 (−6.05, 3.05)	0.0	0.932
Intervention duration (week)				
≤12	4	−6.95 (−20.86, 6.96)	97.0	<0.001
˃12	3	−4.63 (−9.34, 0.09)	0.0	0.667
Probiotic on HbA1c				
Overall	7	−0.11 (−0.18, −0.04)	0.0	0.770
Age (years)				
≤18	1	−0.11 (−0.61, 0.39)	–	–
18−55	4	−0.04 (−0.17, 0.08)	0.0	0.850
˃55	2	−0.14 (−0.23, −0.06)	0.0	0.523
Intervention duration (week)				
≤12	4	−0.09 (−0.17, −0.02)	0.0	0.572
˃12	3	−0.19 (−0.34, −0.03)	0.0	0.947
Probiotic on insulin				
Overall	6	−0.23 (−1.67, 1.20)	0.0	0.961
Age (years)				
≤55	4	−0.22 (−1.93, 1.49)	0.0	0.850
˃55	2	−0.26 (−2.89, 2.37)	0.0	0.642
Intervention duration (week)				
≤12	4	−0.08 (−1.62, 1.45)	79.6	<0.001
˃12	2	−1.27 (−5.30, 2.77)	89.1	<0.001
Probiotic on HDL−C				
Overall	6	2.37 (1.02, 3.71)	0.0	0.849
Intervention duration (week)				
≤12	4	2.29 (0.88, 3.70)	0.0	0.640
˃12	2	3.17 (−1.32, 7.67)	0.0	0.674
Probiotic on LDL−C				
Overall	6	1.98 (−3.19, 7.16)	0.0	0.874
Intervention duration (week)				
≤12	4	2.42 (−3.05, 7.89)	0.0	0.663
˃12	2	−1.71 (−17.68, 14.26)	0.0	0.991
Probiotic on TC				
Overall	5	1.14 (−6.69, 8.98)	14.8	0.320
Intervention duration (week)				
≤12	3	2.96 (−5.39, 11.31)	21.2	0.260
˃12	2	−5.54 (−25.22, 14.13)		
Probiotic on TG				
Overall	6	1.63 (−17.71, 20.97)	55.9	0.045
Intervention duration (week)				
≤12	4	10.40 (−9.87, 30.67)	39.5	0.175
˃12	2	−13.08 (−47.87, 21.71)	44.9	0.178
Probiotic on BMI				
Overall	5	−13.08 (−47.87, 21.71)	0.0	0.998
Intervention duration (week)				
≤12	2	−0.18 (−1.67, 1.31)	0.0	0.939
˃12	3	−0.12 (−1.64, 1.40)	0.0	0.940

### Adverse events

The majority of adverse events were gastrointestinal in nature, including symptoms such as indigestion, abdominal pain, bloating, flatulence, and changes in bowel habits. These adverse events were generally mild, self-limiting, and did not lead to discontinuation of treatment. The reported adverse events showed comparable incidence rates between the intervention and control arms across all six RCTs. Further details are provided in Table 1.

## Discussion

Our comprehensive review revealed that probiotics in patients with prediabetes improved cardiometabolic health, including reduced HbA1c levels and increased HDL-C levels, compared to placebo therapy. However, no significant differences were observed between probiotic supplementation and placebo for other measured parameters, such as FBS, insulin, HOMA-IR, TC, LDL-C, TG, BMI, SBP, and DBP. Furthermore, regarding the safety profile, neither probiotics nor placebo showed significant differences in the occurrence of AEs. The non-significant effects on glycemic, lipid (TG, LDL-C, TC), and obesity (BMI) parameters could imply insufficient dosage/duration, interindividual microbiota differences, or a need for adjunct lifestyle therapies. Heterogeneity of results was high for FBS and TG. While subgroup analyses revealed potential sources of heterogeneity, these findings warrant cautious interpretation. This heterogeneity could stem from differences in study design, population characteristics, probiotics type and dosage, or intervention duration. However, the small number of available studies precluded subgroup analyses for all potential influencing factors. The certainty of the findings was evaluated using the GRADE rating, which was high for HbA1c and HDL-C and moderate for BMI, SBP, DBP, insulin, HOMA-IR, TC, and LDL-C, and low for FBS and TG.

The observed effect of probiotics on HbA1c but not on other glycemic parameters may be attributed to several factors. First, HbA1c reflects average blood glucose over a longer period (2–3 months), while FBS and insulin levels are influenced by short-term factors. Several short-term factors, such as recent meals, timing of food intake, physical activity, stress, and medication adherence, can significantly influence FBS and insulin levels. Furthermore, the low number of studies examining probiotics’ effects on glycemic control and lipid profile in prediabetes limits our understanding of their impact on various markers, making it difficult to generalize findings. The statistical power of our study may also be insufficient to detect small but clinically meaningful changes in fasting glucose or insulin resistance, as well as TG, TC, and LDL-C, which could explain the lack of significant changes in these parameters. Probiotic supplementation can enhance glycolipid control in patients with prediabetes by augmenting HDL-C levels and reducing HbA1c for various reasons. Blood glucose levels are elevated in patients with diabetes and prediabetes due to insulin resistance. Probiotics may decrease insulin resistance by promoting the secretion of glucagon-like peptide-1 (GLP-1) (41). GLP-1 ameliorates insulin resistance by reducing body weight and augmenting the sensitivity of peripheral tissues to insulin (42). The consumption of probiotics results in the synthesis of short-chain fatty acids (SCFAs) in the intestine, which subsequently interact with the G protein-coupled receptor family 43 (GPR43) and GPR41 (43). Inflammatory cytokines play a pivotal role in the pathogenesis of insulin resistance (44). The pro-inflammatory cytokine IL-6 contributes to insulin resistance through serine/threonine phosphorylation of IRS-1, thereby disrupting insulin signal transduction (45). Persistent inflammation is a significant catalyst for insulin resistance, leading to elevated glycosylated hemoglobin levels. Probiotics influence inflammatory responses by directly inhibiting the production of proinflammatory cytokines or indirectly reducing the prevalence of strains associated with proinflammatory processes (46, 47). The administration of probiotics has been demonstrated to substantially decrease the presence of Butyrivibrio crosscuts and *Collinsella aerofaciens*, which are involved in the pro-inflammatory response (38, 48). Probiotics can effectively impede the progression of insulin resistance by enhancing blood lipid levels. Probiotic supplementation demonstrates hepatoprotective effects against hypercholesterolemia-induced damage by downregulating gluconeogenic enzyme expression while upregulating glycogen synthase genes in hepatic tissue (49).

Although probiotic supplementation was associated with statistically significant improvements in HbA1c and HDL-C levels, it is not entirely clear which trials contributed most strongly to these effects. Some RCTs reporting substantial changes in these outcomes appeared to have relatively small sample sizes and higher standard deviations, suggesting that studies with less precision may have disproportionately influenced the pooled effect estimates (35, 37). This raises the possibility of small-study effects or publication bias, even in the presence of low statistical heterogeneity. Although the direction and magnitude of the effects were consistent, the robustness of these findings may still be limited by methodological variability and potential confounders not adjusted for in the primary studies. Future meta-analyses should consider influence diagnostics and sensitivity analyses to determine the extent to which individual studies affect overall estimates.

The findings of our investigation align with the meta-analysis mentioned above by Li et al. (20). Based on the findings, they suggested probiotics could provide metabolic advantages in prediabetes management by improving HbA1c and lipid parameters. Significant differences are evident between the previous meta-analysis by Li et al. (20) and our current meta-analysis. The initial distinction pertains to the number of studies included in the analysis. Li et al. (20) included only seven RCTs. Of the seven RCTs, one was published in Chinese and was excluded from prominent international databases. Thus, the validity and accuracy of its contents are uncertain. Our current meta-analysis includes 10 RCTs published in English and indexed in international databases. The second distinction is the inclusion of a greater number of outcomes compared to the previous meta-analysis by Li et al. (20) We specifically analyzed the BMI, SBP, HOMA-B, and DBP changes from the initial measurements not addressed in the previous study. The third difference is the inconsistent data entry by Li et al. (20), who calculated the HbA1c and HOMA-IR outcomes by subtracting baseline values from follow-up measurements in probiotic and placebo groups. Conversely, for other outcomes, including FBS, LDL-C, TC, HDL-C, and TG, only the values obtained during the follow-up period were recorded, without subtracting the baseline values from the follow-up values.

The strengths of this study include a rigorous methodology, adherence to PRISMA guidelines, and a comprehensive GRADE assessment to evaluate the certainty of evidence. We ensured robust and generalizable findings by using a random-effects model and conducting subgroup and sensitivity analyses. Additionally, including trials from diverse geographical locations enhances the external validity of our results. There were some limitations that must be mentioned. First, the relatively small number of included studies and participants, along with heterogeneity in probiotic strains, dosages, and formulations, may have impacted the reliability of the findings in this meta-analysis. Second, inadequate studies for gender-specific analysis. Third, most of the included trials did not account for potential confounding factors such as dietary habits, physical activity levels, smoking status, and other lifestyle-related variables that may influence cardiometabolic outcomes in individuals with prediabetes. Fourth, the non-significant glycemic and lipid profile changes might indicate that longer supplementation periods or higher dosages are needed to observe measurable outcome changes. Consequently, further well-designed RCTs are required to establish robust clinical evidence.

## Conclusion

This systematic review and meta-analysis demonstrate that probiotics supplementation can somewhat improve cardiometabolic health features by substantially decreasing HbA1c levels and increasing HDL-C levels in individuals with prediabetes. Moreover, probiotics did not have a significant effect on FBS, fasting insulin, HOMA-IR, TC, LDL-C, TG, BMI, SBP, and DBP. Further studies are needed to determine the benefits of probiotics on patients with prediabetes. GRADE assessment showed high for HbA1c and HDL-C and moderate for BMI, SBP, DBP, insulin, HOMA-IR, TC, and LDL-C, and low for FBS and TG.

## Data Availability

The datasets presented in this study can be found in online repositories. The names of the repository/repositories and accession number(s) can be found in the article/Supplementary material.
