# Supplementary material for: The effects of probiotic supplementation on cardiometabolic health in patients with prediabetes: a systematic review, meta-analysis, and GRADE assessment
Source: Front Nutr. 2025 Sep 1;12:1616476. doi: 10.3389/fnut.2025.1616476 (PMC12434957; doi:10.3389/fnut.2025.1616476)
Supplement: Supplementary file 1 [file Data_Sheet_1.PDF]

Supplementary Table 1. Search strategy

Intervention	outcome	RCT
probiotic<br>probiotics<br>Lactobacillus<br>Bifidobacterium	prediabetes<br>pre-diabetes<br>prediabetic state<br>impaired fasting glucose<br>IFG<br>impaired glucose tolerance<br>IGT	"Randomized Controlled Trial" OR intervention OR "Controlled Clinical Trial" OR random OR placebo OR assignment OR "controlled trial" OR "Clinical Trial" OR trial

	Search strategy
Pubmed	("probiotic"[MeSH Terms] OR " probiotic"[Title/Abstract] OR " probiotics "[Title/Abstract] OR " Lactobacillus "[Title/Abstract] OR " Bifidobacterium "[Title/Abstract])AND ("prediabetes"[MeSH Terms] OR " prediabetes"[Title/Abstract] OR " pre-diabetes"[MeSH Terms] OR " prediabetic state"[Title/Abstract] OR " impaired fasting glucose"[Title/Abstract] OR "IFG"[Title/Abstract] OR "impaired glucose tolerance"[Title/Abstract] OR "IGT"[Title/Abstract]) AND ("Randomized Controlled Trial"[Publication Type] OR "Randomized Controlled Trial"[Title/Abstract] OR "intervention"[Title/Abstract] OR "Controlled Clinical Trial"[Publication Type] OR "Controlled Clinical Trial"[Title/Abstract] OR "random"[Title/Abstract] OR "placebo"[Title/Abstract] OR "assignment"[Title/Abstract] OR "controlled trial"[Title/Abstract] OR "Clinical Trial"[Publication Type] OR "Clinical Trial"[Title/Abstract] OR "trial"[Title/Abstract])
Scopus	(TITLE-ABS-KEY("probiotic" OR "probiotics" OR "Lactobacillus" OR "Bifidobacterium") AND TITLE-ABS-KEY("prediabetes" OR " pre-diabetes" OR " prediabetic state" OR " impaired fasting glucose" OR " IFG " OR " impaired glucose tolerance" OR " IGT") AND TITLE-ABS-KEY("Randomized Controlled Trial" OR intervention OR "Controlled Clinical

	Trial" OR random OR placebo OR assignment OR "controlled trial" OR "Clinical Trial" OR trial))
ISI web of science	"probiotic" OR "probiotics" OR "Lactobacillus" OR "Bifidobacterium" (Topic) and ("prediabetes" OR " pre-diabetes" OR " prediabetic state" OR " impaired fasting glucose" OR " IFG " OR " impaired glucose tolerance" OR " IGT" (Topic) and "Randomized Controlled Trial" OR intervention OR "Controlled Clinical Trial" OR random OR placebo OR assignment OR "controlled trial" OR "Clinical Trial" OR trial (Topic)

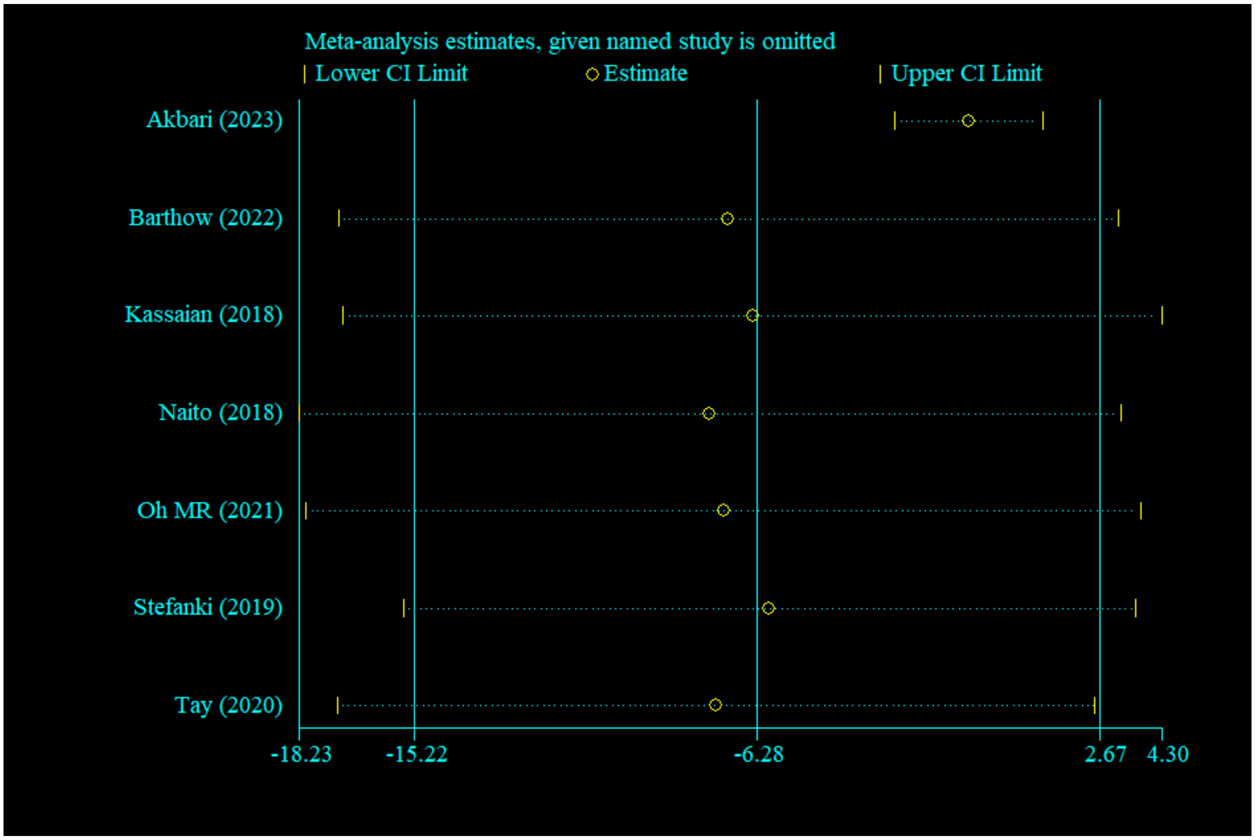

Figure S1. Sensitivity analysis for FBS

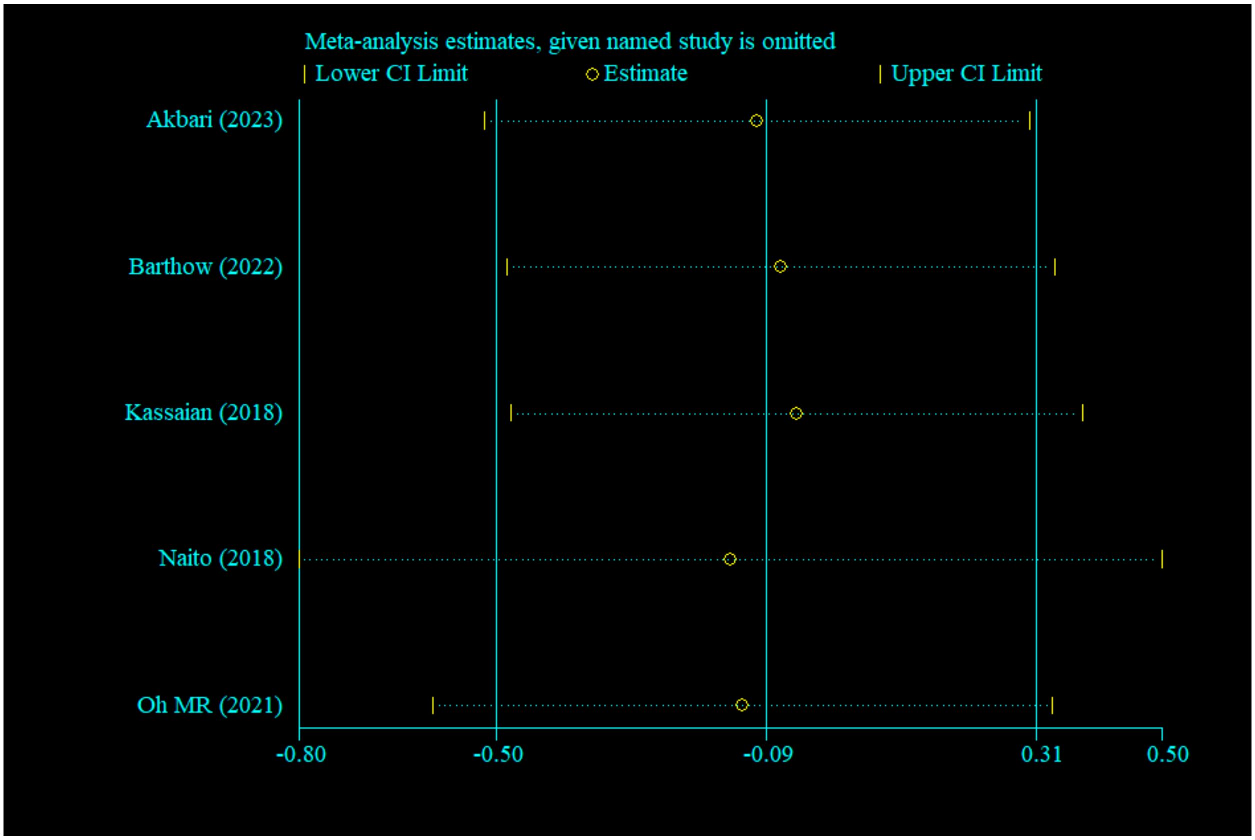

Figure S2. Sensitivity analysis for HOMA-IR

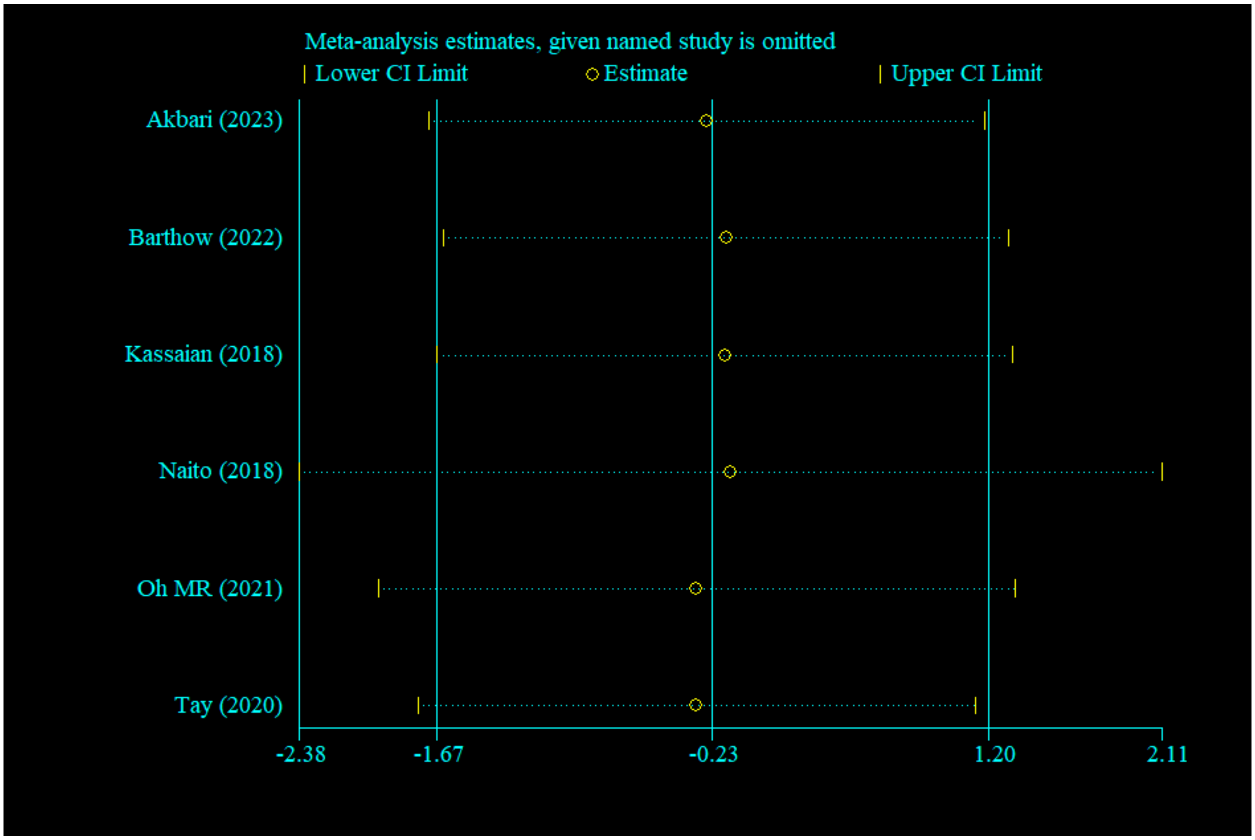

Figure S3. Sensitivity analysis for insulin.

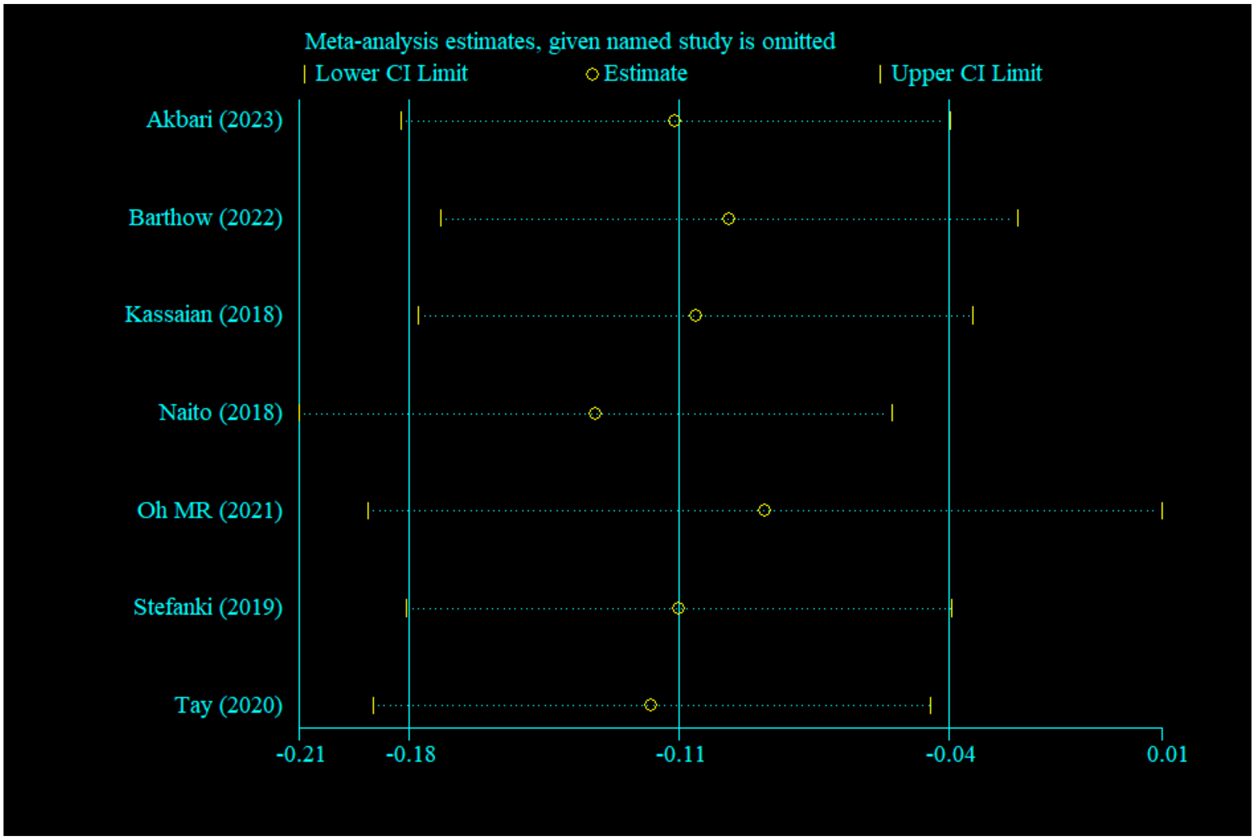

Figure S4. Sensitivity analysis for HbA1c

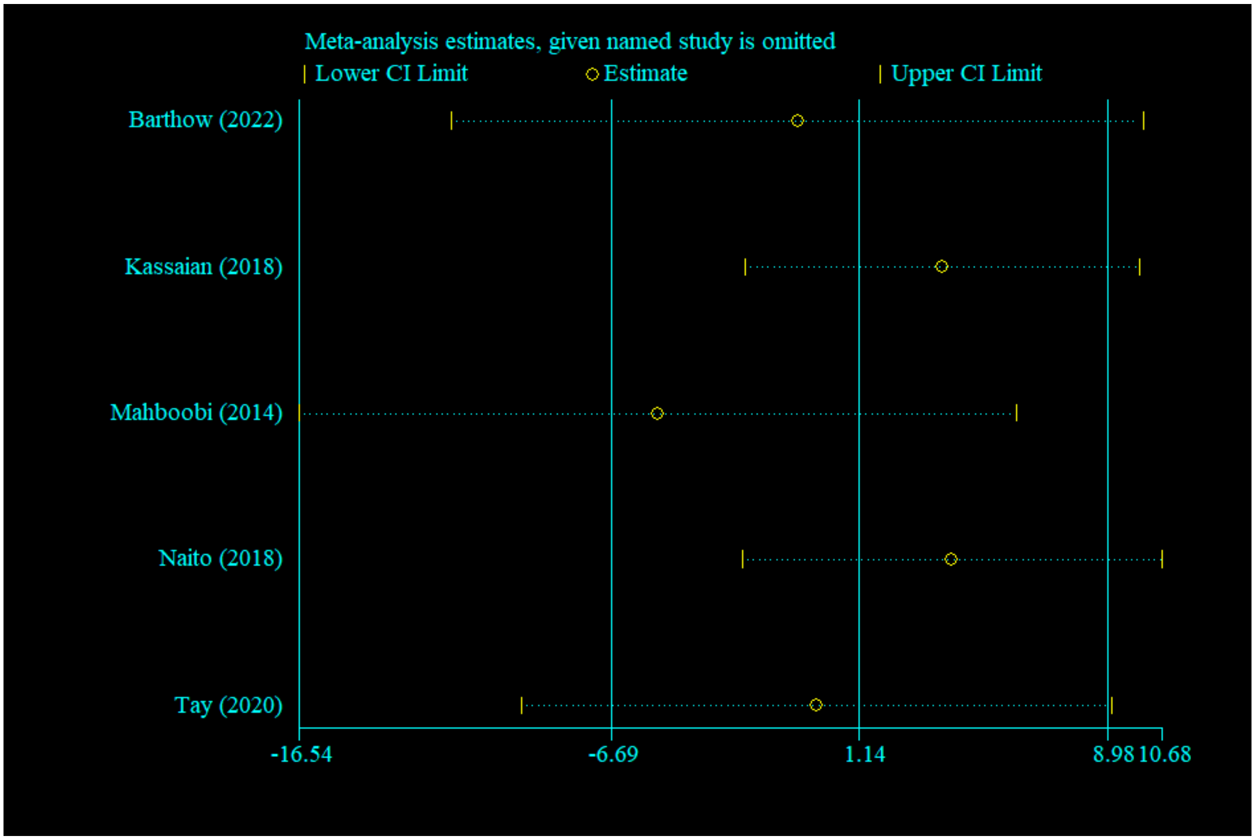

Figure S5. Sensitivity analysis for TC.

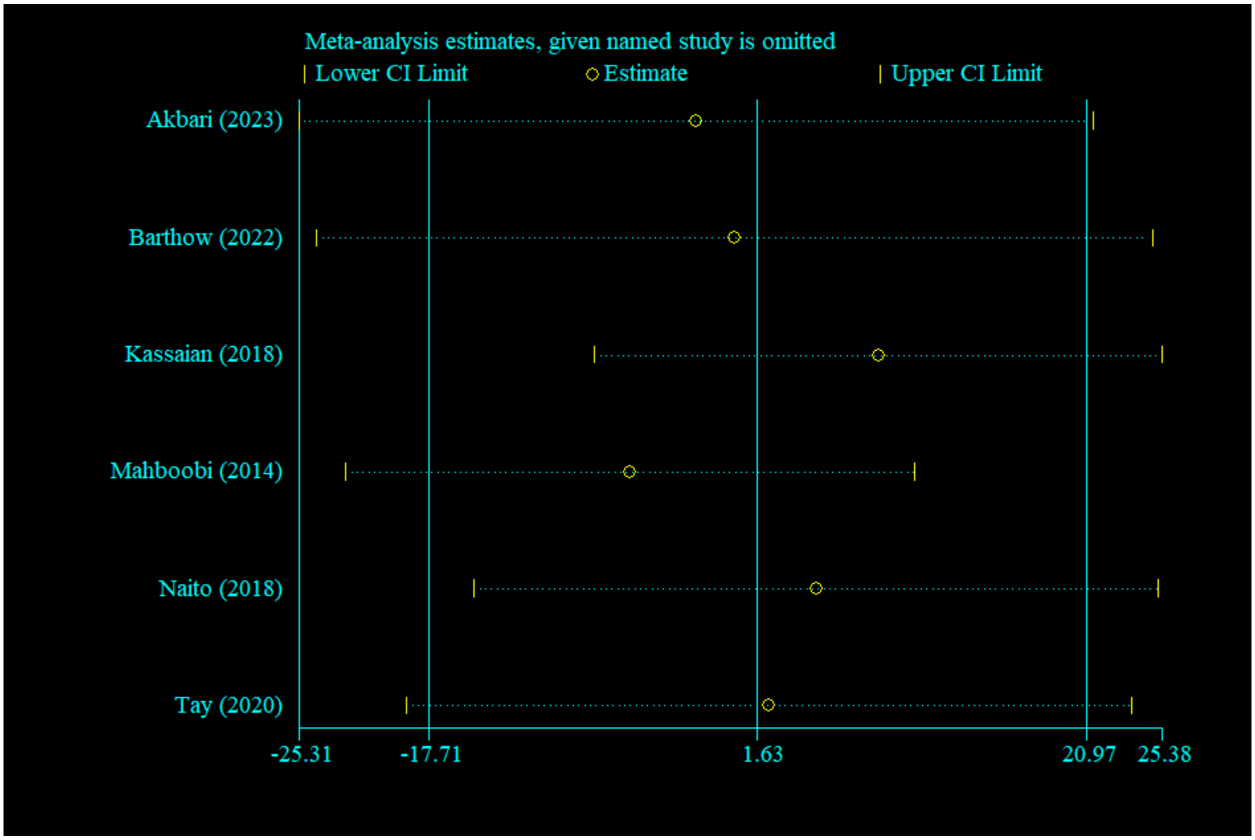

Figure S6. Sensitivity analysis for TG.

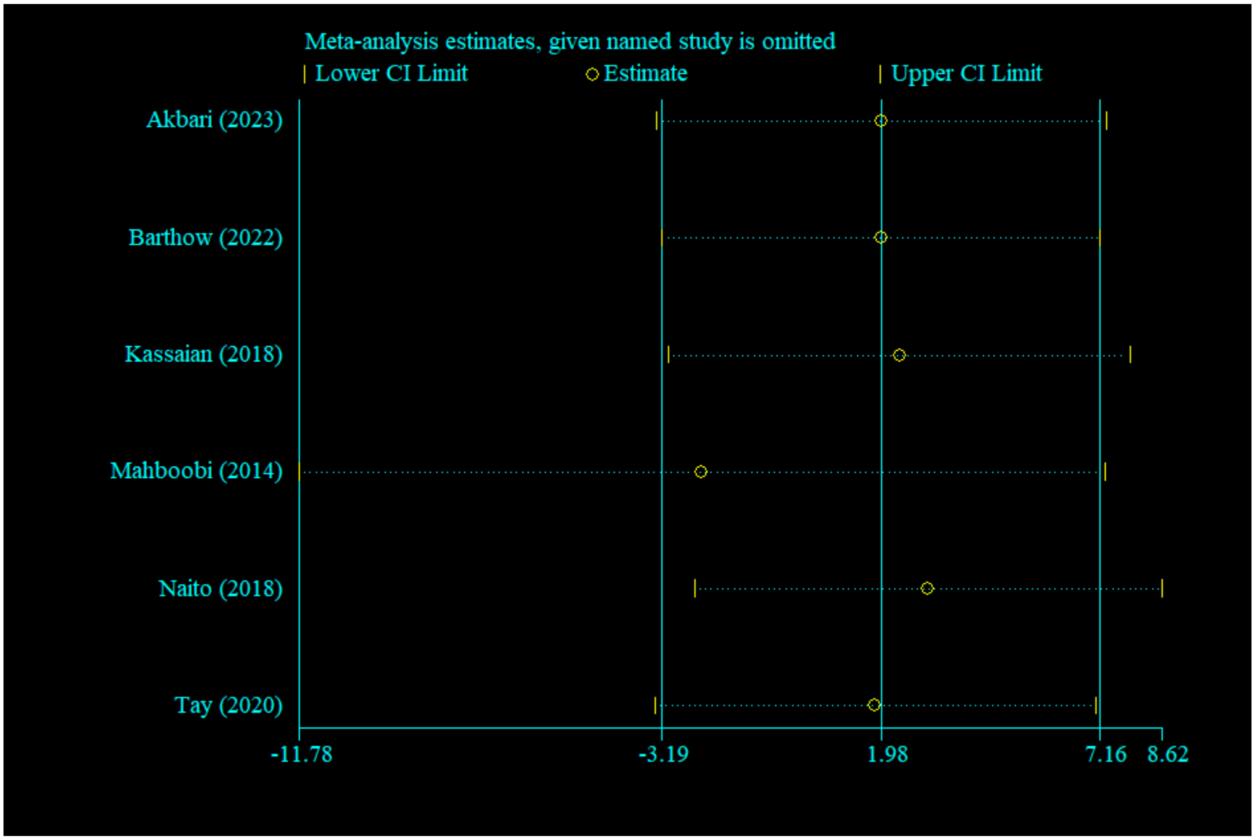

Figure S7. Sensitivity analysis for LDL-C.

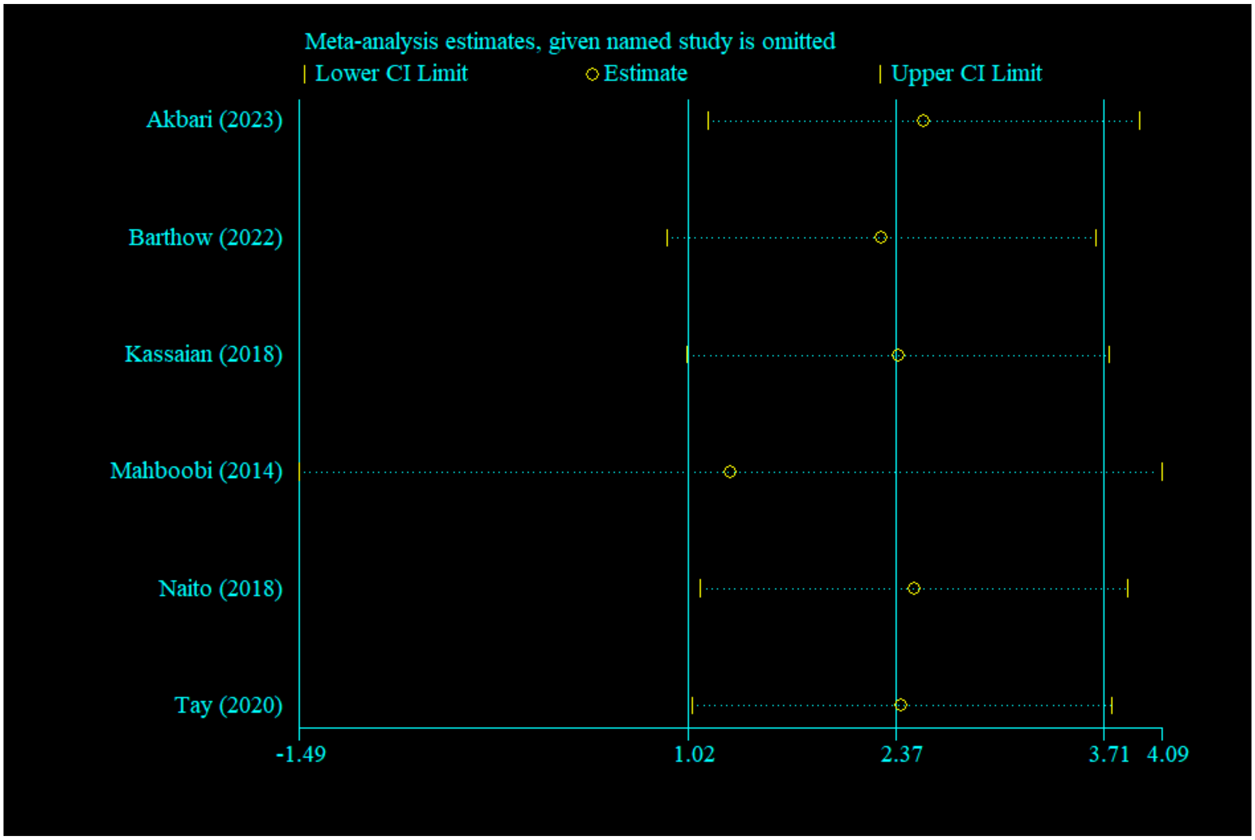

Figure S8. Sensitivity analysis for HDL-C.

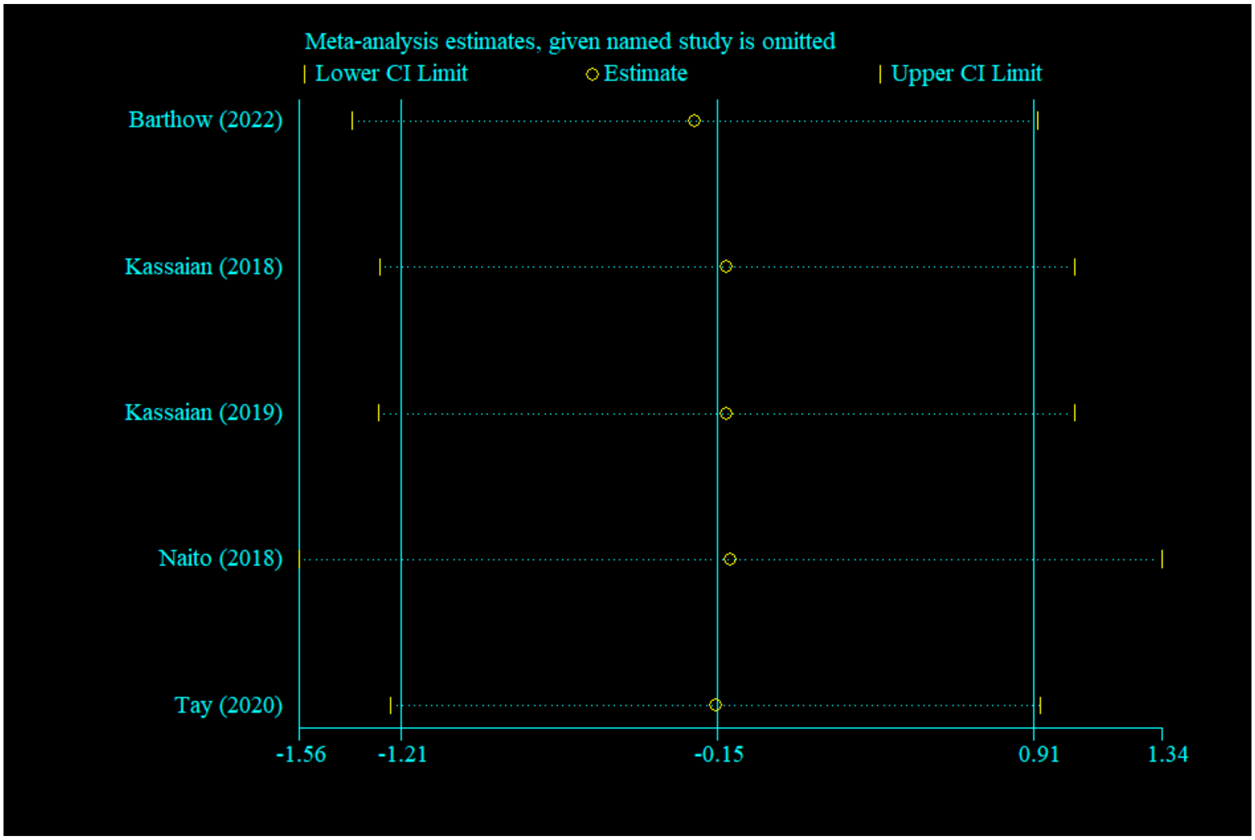

Figure S9. Sensitivity analysis for BMI.

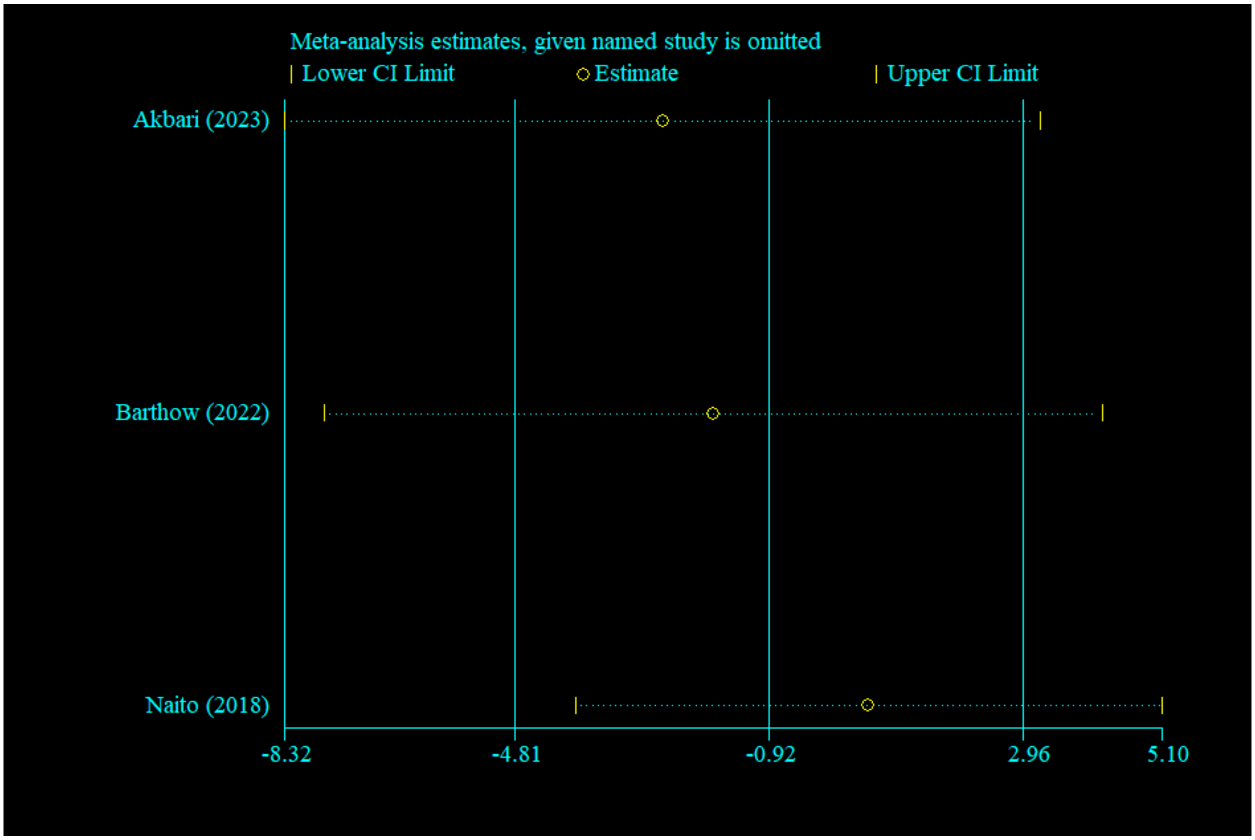

Figure S10. Sensitivity analysis for SBP.

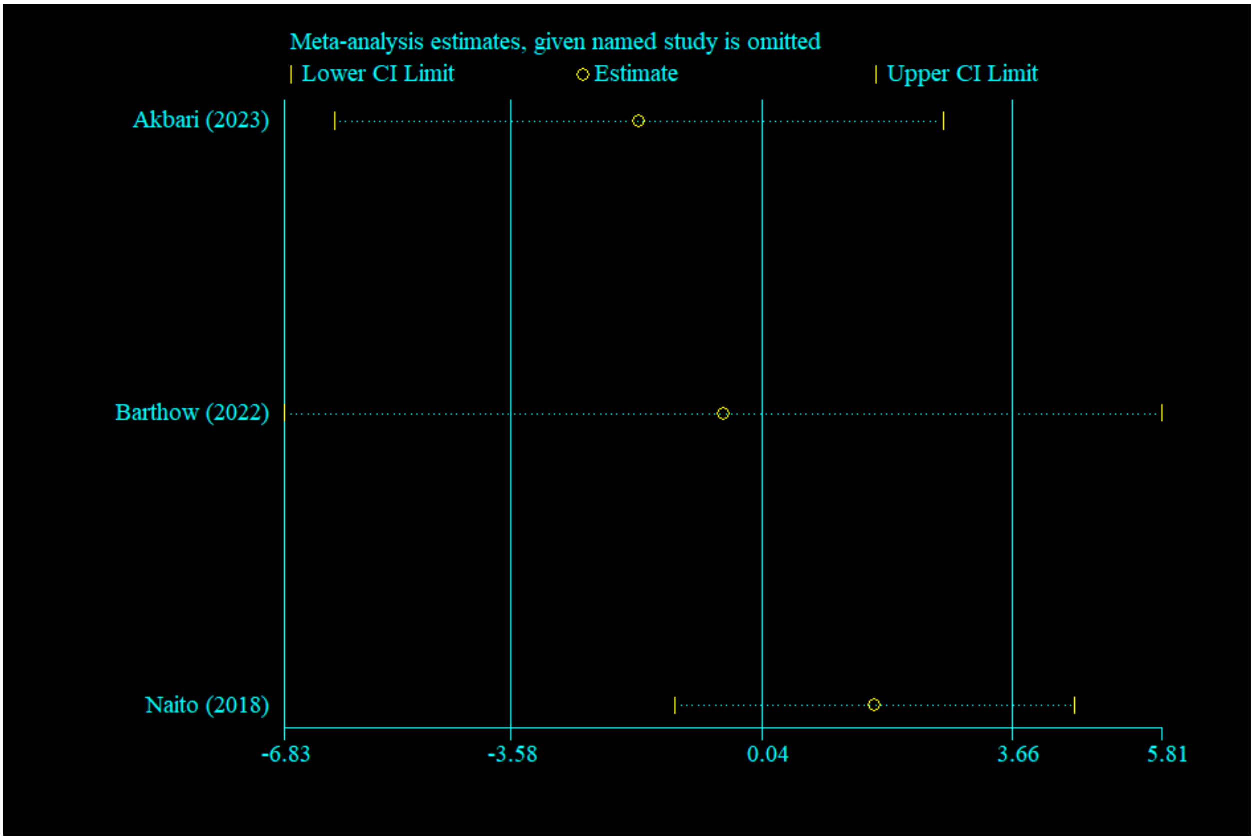

Figure S11. Sensitivity analysis for DBP.
